# Pediatric magnetoencephalography

**DOI:** 10.1002/cns3.20011

**Published:** 2023-02-03

**Authors:** Douglas R. Nordli, Fernando N. Galan

**Affiliations:** ^1^ Department of Child and Adolescent Neurology, Mayo Clinic College of Medicine and Health Sciences Jacksonville Florida USA; ^2^ Department of Child and Adolescent Neurology, Nemours Children's Health Jacksonville Florida USA

**Keywords:** epilepsy, magnetoencephalography, pediatrics

## Abstract

Magnetoencephalography (MEG) is a technology used in pediatric and adult epilepsy that records magnetic fields produced from electric currents in the brain. MEG can locate epileptogenic zone(s), lateralize language functions, localize sensorimotor cortex, and identify visually evoked fields. It is a powerful technology with key advantages in pediatrics. The majority of its limitations are resource driven. With advancing technology, MEG will become a more prominent and valuable tool used in pediatric epilepsy and epilepsy surgery in the future. We review MEG and provide illustrative cases to showcase its usage.

## Introduction

Approximately 7%–20% of all pediatric patients diagnosed with epilepsy will follow a drug‐resistant course.^1^ The associated morbidity, mortality, potential for developmental regression, and loss of quality of life pose major challenges for both parents and clinicians to manage. Epilepsy surgery for many patients remains the only hope of cure or significant reduction in seizure frequency and severity. The noninvasive tests used in planning and performing epilepsy surgery help guide the ability to obtain an adequate resection of diseased tissue while sparing a healthy brain. The importance of reliable and accurate technology is paramount. Magnetoencephalography (MEG) is a technology that records magnetic fields produced from electric currents in the brain.^2^ Its use has grown given its ability to locate epileptogenic zones, lateralize language functions, localize sensorimotor cortex, and identify visually evoked fields.^3^ This review highlights the uses of MEG through example cases in pediatric drug‐resistant epilepsy and epilepsy surgery.

### History and design concept

MEG was discovered in 1968 by David Cohen. MEG technology is largely adapted from a similar technology—called magnetocardiography—that detects magnetic fields in the heart. MEG samples magnetic fields using superconducting quantum interference devices (SQUIDs).^4^ A SQUID is a device made up of superconducting loops containing Josephson junctions, which measure magnetic fields.^5^ Initially a single SQUID was moved across a patient's scalp to query independent regions. Later, SQUIDs were arranged in arrays to cover a larger surface area of the scalp, thereby making the technology fundamentally more user‐friendly. MEG samples 3–4 cm^2^ of cortical electric activity by placing 306 sensors across the brain.^4^ It detects magnetic fields generated by intracellular currents predominantly from dendritic cells in contrast to electroencephalography (EEG), which detects extracellular currents predominantly from pyramidal cells.^6^

MEG has been utilized in the world of pediatric epilepsy surgery for only the past three decades or so. Given the paucity of MEG machines in the world, pediatric neurologists and epileptologists may not be universally exposed to its powers.

### Utility

MEG's ability to locate epileptogenic zones and eloquent cortices hinges upon its ability to solve inverse problems.^7^ An inverse problem works by collecting observations and then calculating the causal factors that produced the given observations. The areas of interest in a MEG study—like the eloquent cortex—are separated from the brain's normal background electric activity. This is accomplished through magnetic source imaging (MSI). MSI uses fiducial points to identify activated brain regions.^8^ Fiducial points are reference points that are typically placed in three locations on a patient's head (left pre‐auricular, right pre‐auricular, and nasion). These points are used to create a map of the brain and magnetometers in space.^8^ Fiducial points use lipid markers as reference points on magnetic resonance imaging (MRI).^8^ The structural magnetic resonance image is then co‐registered with the MEG recording data to create the magnetic source image. This ultimately provides MEG the ability to identify language centers, motor cortex, and sensory cortex.

MEG and EEG have been studied and are complementary studies. Over 50% of patients have interictal epileptiform discharges (IEDs) that are visible on both EEG and MEG.^9^ Seven percent of IEDs may be visible only on EEG compared to 18% of IEDs that may be identified only by MEG.^9^ The IEDs that can be seen on MEG but not EEG are often from deep tissue in the brain such as the frontal lobe, temporal lobe, and insula.^10^ Classically, these epilepsies can be difficult to diagnose in children as the scalp EEG may be negative. The classic semiology of these seizure types is often absent as children—especially young children—struggle to report internal focal features prior to generalization, which detracts from the physician's ability to localize based on semiology.

MEG's ability to identify IED where EEG cannot has direct implications in planning the presurgical evaluation of potential epilepsy surgery candidates. This may be related to the superior coverage that MEG offers with its 306 sensors that cover the entire brain. MEG has been shown to be superior to conventional scalp EEG and high‐density EEG in localizing interictal discharges.^11^ Identifying the irritative zone helps epileptologists and neurosurgeons plan surgeries with good hypotheses in order to create the best possible blueprint to obtain meaningful information from invasive recording. MEG can be particularly helpful in cases of frontal lobe epilepsy that masquerade as a generalized epilepsy on the scalp EEG recording.^12^ It also has distinct advantages in identifying seizures of deep tissue onset.

MEG is useful in identifying a single epileptogenic zone in patients who have multiple MRI lesions.^10^, ^13^ MRI‐negative patients may benefit from MEG given its ability to localize clusters of discharges that may identify MRI‐negative cortical dysplasias.^10^, ^14^

Studies have shown a high concordance between interictal MEG localization and localization data obtained from intracranial EEG recording.^15^ MEG's power is not confined only to IEDs. Ictal MEG has been shown to have superior lateralizing and localizing power compared to scalp EEG in identifying the seizure onset zone.^16^, ^17^

MEG systems like the BabySQUID and BabyMEG are used for infants and toddlers with drug‐resistant epilepsy.^18^ These systems allow for improved data collection because the sensors can be positioned closer to the brain and fit more easily to young patients, which aids in ease of setup.^18^

### Limitations

The major limitations to the utility of MEG are resource driven. Estimates indicate that there are likely no more than 200–300 machines in the world.^19^ The cost of building and operating MEG is significant and thus it may not be feasible for centers to universally possess. MEG requires a specialized room free of magnetic noise to reduce or eliminate artifacts. SQUIDs require very low temperatures to record accurately, and the cost of maintaining a MEG is high.^20^ MEG sensors are not fixed to the scalp of the patient and are thus susceptible to motion artifacts. The ability to operate a MEG and analyze patients relies on a team of highly trained MEG professionals. Currently, few training programs in the country exist, thereby restricting the number of trained professionals.

### Future of MEG

MEG technology continues to advance. As technology in general advances, the cost of production and distribution of complex machines decreases. MEG is no exception. Models predict that future MEGs may be five times less expensive, which would increase the ability of medical centers to purchase and operate this technology.^4^ Advances are also being made to produce a mobile MEG, which would increase patient access. Optically pumped magnetometers (OPMs) show promise as a wearable alternative that is similar to MEG. OPMs are quantum sensors that measure magnetic fields through the manipulation of a quantum property called spin.^21^ A particle's movement in response to a magnetic field is its spin. OPMs use a light source to measure spin as a reflection of the underlying magnetic field. A major advantage of OPMs compared to MEG is that OPMs do not rely on cryogenic cooling and thus can be placed within millimeters of the patient's scalp.^21^ With such close proximity, OPMs are more sensitive to smaller magnetic fields.^21^ Researchers in the MEG field continue to work to find more accurate information identifying the seizure onset zone in patients with epilepsy. Recently, a new phenomenon called the ripple onset zone (ROZ) has been shown to have a high localizing value to the seizure onset zone.^22^ The ROZ better understands the propagation of electricity to better estimate the epileptogenic zone.^22^ Further research is needed in this area, but preliminary studies have shown promise in epilepsy surgery planning. High‐frequency oscillations (HFOs) have been shown to be superior to sharp waves in identifying the seizure onset zone in patients.^23^ These HFOs have also been studied with MEG. There is early evidence to suggest that HFO mapping with MEG may be helpful for mapping epilepsy surgery, although further research is needed.^23^ Studies pairing modalities such as MEG and functional MRI show promise in more accurate localization of seizure onset zone. Finally, MEG use continues to expand not only in epilepsy but also in other diseases like dementia, autism, anxiety, and depression.^24^, ^25^, ^26^

## Example Cases

### Case 1

**Figure 1:**
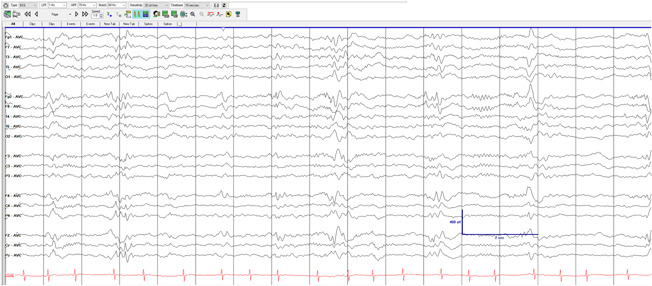
Average referential montage showcasing right frontal and central maximum sharp waves.

**Figure 2:**
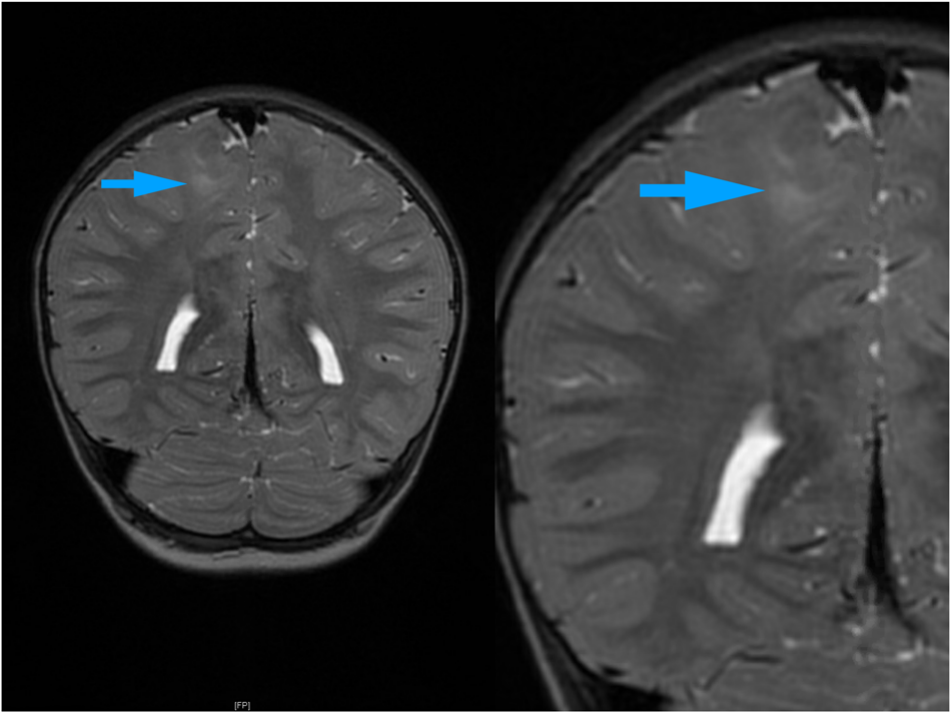
Post‐contrast coronal T2 magnetic resonance imaging with hyperintense signal in the right superior frontal gyrus (blue arrow).

A 20‐month‐old boy presented to the pediatric tertiary care hospital with episodic independent right and left tonic arm extension and L‐predominant clonic activity. These events occurred one to two times per day. His initial EEG (Figure 1) revealed midline and right frontal maximal sharp waves. His MRI revealed a large region of focal cortical dysplasia in the right superior frontal gyrus (Figure 2). The location of the dysplasia was potentially concerning as it was near the motor cortex. As the patient grew older, his seizures became drug resistant. He failed multiple medication trials, prompting presurgical evaluation. MEG was performed to better understand the epileptogenic zone and its relationship to the eloquent cortex and to determine if he would be a candidate for a proposed surgical intervention.

**Figure 3:**
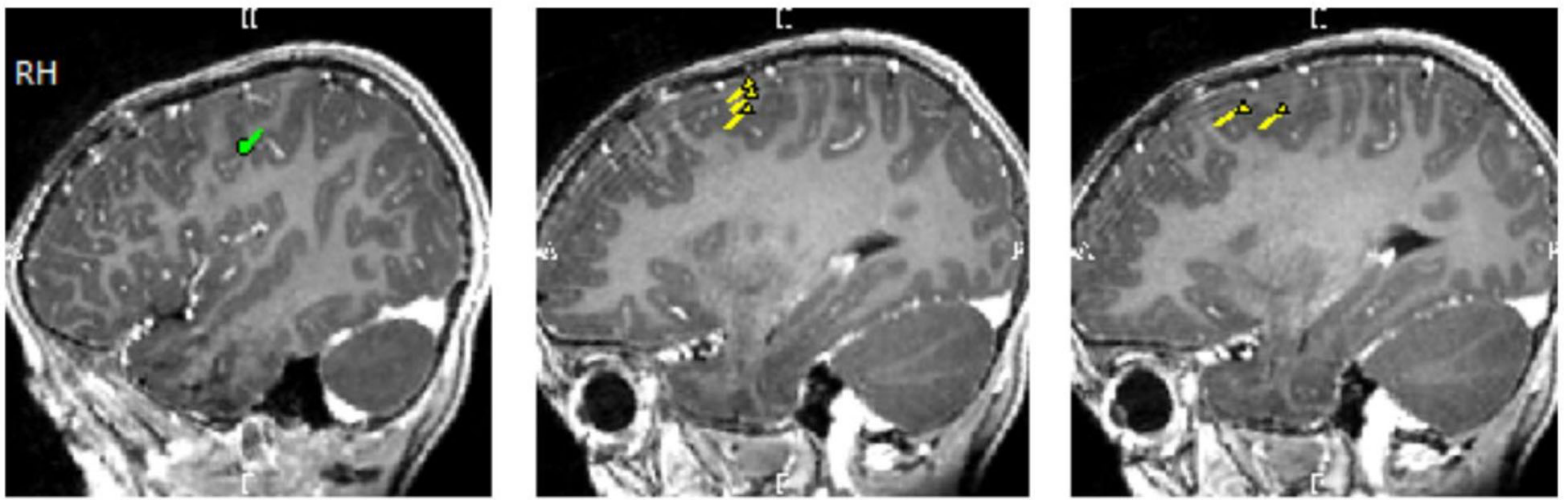
Magnetoencephalography images. Yellow arrows indicate interictal discharges. Green arrows indicate somatosensory responses.

Case 1 highlights MEG's ability to densely localize an epileptogenic zone in addition to eloquent motor cortex in a very young pediatric patient. Given the patient's age, other diagnostic modalities such as functional MRI and transmagnetic stimulation would not be well tolerated. The MEG clearly localized the epileptogenic zone (Figure 3) in addition to the eloquent motor cortex, allowing plans to resect the lesion to proceed.

### Case 2

**Figure 4:**
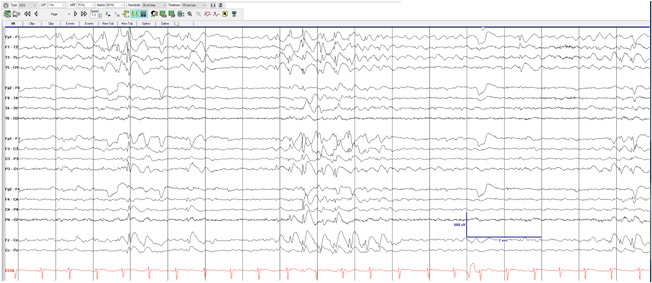
Longitudinal bipolar montage with left temporal sharp wave complexes and intermixed slowing.

**Figure 5:**
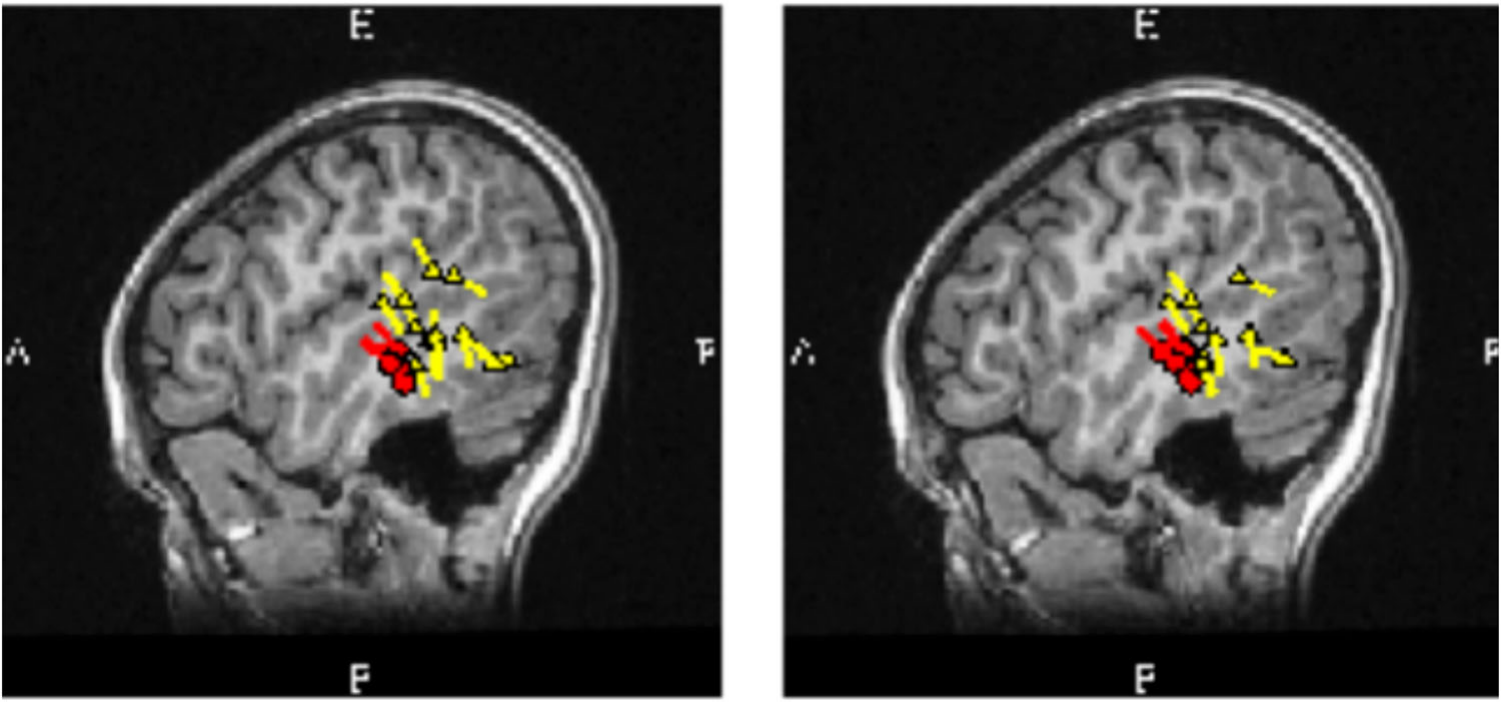
Magnetoencephalography images. Yellow arrows indicate interictal discharges. Red arrows indicate receptive language.

A nine‐year‐old girl presented to the tertiary care children's hospital with new onset seizures. The first event was generalized in semiology. An EEG performed was abnormal due to the presence of left temporal sharp waves and slowing (Figure 4). 3T MRI was normal. After the addition of levetiracetam, the patient's seizure semiology changed and she experienced frequent episodes of right head turn, right eye deviation, and right facial and hemibody clonic activity with loss of consciousness. Given the refractory nature of her seizures, surgical candidacy was explored. As a part of the evaluation, MEG was performed (Figure 5).

Case 2 highlights MEG's ability to localize potentially MRI‐negative areas of cortical dysplasia and receptive language. This advantage allows epileptologists to better counsel families about further options—such as surgery—and the associated risks and benefits.

## Conclusion

As a diagnostic and surgical planning tool for pediatric patients with drug‐resistant epilepsies, MEG has clear advantages and few limitations. As the cost of MEG decreases, it should become increasingly available in comprehensive epilepsy centers. Pediatric epilepsy specialists should be urged to familiarize themselves with this powerful tool in order to provide patients with the best care and the highest chance of achieving seizure freedom.

## Author Contribution

**Douglas R. Nordli III**: Conceptualization; data curation; writing – original draft. **Fernando N. Galan**: Conceptualization; formal analysis; writing – review and editing.

## Conflict of Interest

The authors declare no conflict of interest.
